# Imaging-Based Biomarkers of Cognitive Performance in Older Adults Constructed via High-Dimensional Pattern Regression Applied to MRI and PET

**DOI:** 10.1371/journal.pone.0085460

**Published:** 2013-12-31

**Authors:** Ying Wang, Joshua O. Goh, Susan M. Resnick, Christos Davatzikos

**Affiliations:** 1 Section of Biomedical Image Analysis, Department of Radiology, University of Pennsylvania, Philadelphia, Pennsylvania, United States of America; 2 Laboratory of Behavioral Neuroscience, National Institute on Aging, Baltimore, Maryland, United States of America; 3 Graduate Institute of Brain and Mind Sciences, National Taiwan University College of Medicine, Taipei, Taiwan; Banner Alzheimer's Institute, United States of America

## Abstract

In this study, we used high-dimensional pattern regression methods based on structural (gray and white matter; GM and WM) and functional (positron emission tomography of regional cerebral blood flow; PET) brain data to identify cross-sectional imaging biomarkers of cognitive performance in cognitively normal older adults from the Baltimore Longitudinal Study of Aging (BLSA). We focused on specific components of executive and memory domains known to decline with aging, including manipulation, semantic retrieval, long-term memory (LTM), and short-term memory (STM). For each imaging modality, brain regions associated with each cognitive domain were generated by adaptive regional clustering. A relevance vector machine was adopted to model the nonlinear continuous relationship between brain regions and cognitive performance, with cross-validation to select the most informative brain regions (using recursive feature elimination) as imaging biomarkers and optimize model parameters. Predicted cognitive scores using our regression algorithm based on the resulting brain regions correlated well with actual performance. Also, regression models obtained using combined GM, WM, and PET imaging modalities outperformed models based on single modalities. Imaging biomarkers related to memory performance included the orbito-frontal and medial temporal cortical regions with LTM showing stronger correlation with the temporal lobe than STM. Brain regions predicting executive performance included orbito-frontal, and occipito-temporal areas. The PET modality had higher contribution to most cognitive domains except manipulation, which had higher WM contribution from the superior longitudinal fasciculus and the genu of the corpus callosum. These findings based on machine-learning methods demonstrate the importance of combining structural and functional imaging data in understanding complex cognitive mechanisms and also their potential usage as biomarkers that predict cognitive status.

## Introduction

 Aging is associated with declines in neurocognitive functions often characterized by degeneration of brain tissue, changes in brain function, and accelerated cognitive decline, particularly in clinical cases such as Alzheimer's disease (AD). However, despite the connection with neural changes, diagnosis of clinical cognitive impairment to date still primarily depends on cognitive changes measured by neuropsychological assessments. Neuroimaging studies have shown that structural and functional brain changes often precede clinical symptoms of cognitive impairment [1], which resulted in the revision of clinical diagnostic criteria for mild cognitive impairment (MCI) and AD [2]. Thus, neuroimaging analysis based on machine learning techniques provide an important means to understand the nature of brain-cognition associations and identify the most diagnostic imaging biomarkers for early detection of individuals at risk for cognitive impairment.

Finding specific brain regions involved in a cognitive process has been a recurrent goal in functional neuroimaging in the last two decades [3-5]. In these studies, univariate generalized linear modeling (GLM) has been widely used to create statistical maps of voxels sensitive to a cognitive task above some particular threshold [6-8]. Apart from voxel-based GLM analysis, some studies also examine relationships between predefined regions of interest (ROIs) and cognitive task performance [4,5]. However, these univariate and ROI-based methods lack sufficient sensitivity and specificity for effective analysis of complex neurocognitive phenomena, which might involve covariation of activities between networks of brain regions that have yet to be defined *a priori*. As such, characterizations of brain changes in normal or clinical aging have had specific and limited scopes within each study and have been relatively unwieldy in the context of the multi-faceted nature of the issue.

To overcome the limitations of univariate and ROI-based approaches, machine learning-based pattern analysis methods have been widely adopted to automatically derive distinctive brain regions related to a hypothesis or a given study objective [9]. The majority of pattern analysis studies focused on categorically classifying individuals as either healthy or diseased [1,10-14]. More recently, pattern regression methods have addressed the need for measuring continuous and graded stages of brain markers associated with disease development and cognitive performance [15-20]. These methods not only capture the general nature of disease development, they also take into account individual variance in brain tissue changes, and are thus able to provide diagnostic or prognostic imaging biomarkers to detect people in pre-clinical stages of brain disease, so that early treatment intervention can begin. These advanced pattern analysis methods can also be readily applied on combined imaging data modalities to improve the detection of changes in brain structure and function before any measurable cognitive changes using neuropsychological assessments [1,2]. Whereas a single imaging modality provides only partial information (e.g. only structural volumes), multiple imaging modalities (e.g. structural and functional brain images) provide richer and complementary information to discovering brain regions associated with a particular brain disease [21,22]. Although multiple-modality-based analysis has been widely used to support clinical disease diagnosis [23-25], few studies have applied them to quantify the brain changes associated with cognitive domains in normal aging. This present study aims to evaluate imaging biomarkers showing brain differences related to continuous and subtle cognitive performance differences across normal individuals by using combined imaging modalities. 

We extended a previous single-modality pattern regression method [19] to multi-modal imaging data to identify structural and functional imaging biomarkers of cognitive ability, which was represented by continuous cognitive scores. We focused on two cognitive domains known to decline with aging: memory, specifically long-term and short-term memory, and executive processing, specifically semantic retrieval and manipulation [26,27]. While details of our pattern regression method have been reported in our previous study [19], there are some important features worth highlighting briefly here. First, by adopting adaptive feature extraction and sparse relevance vector machine (RVM) regression algorithms, our machine learning methodology has good generalizability to characterize various relationships between brain images and clinical variables, including different measures of cognition. Notably, whereas classical statistical methods focus more on parameter significance to make conclusions regarding *a priori* hypotheses [28], machine learning based models such as ours are validated directly by their predictive performance with respect to the target variable. Second, we use voxel clustering and recursive feature elimination (RFE) algorithms on all imaging modalities to adaptively select brain regions that contribute the most distinctive information that are then used to train and optimize our relevance vector regression (RVR) model. Third, the use of RVR analysis on brain data provides a continuous diagnostic/prognostic score rather than a categorical classification to estimate individual cognitive performance on a given cognitive function [19,29]. Finally, our method also enables us to directly compare differential contributions of gray and white matter structural MRI images and PET images of regional cerebral blood flow (rCBF) to memory and executive functions having obtained the optimal RVR model for each case. We expected that multi-modal imaging data should be more informative than single-modalities in accounting for cognitive performance. Moreover, different brain regions would show dissociable contributions to specific cognitive domains, reflecting differential importance of each brain region in a process-specific manner [30].

## Materials and Methods

### Participants

The present study used baseline MRI and PET-rCBF scans from 132 older adult participants (baseline mean age (SD) = 69.59 (7.75) yrs; male/female: 78/54) from the Baltimore Longitudinal Study of Aging (BLSA) neuroimaging sub-study [31,32] who remained cognitively normal throughout the ongoing longitudinal study. The BLSA neuroimaging study is a prospective study investigating structural, functional, and cognitive changes associated with normal aging and cognitive impairment [32]. Annual or semi-annual longitudinal imaging and clinical evaluations were acquired, but only baseline scans and baseline cognitive performances of participants were applied in this present work. Cognitive status was determined using consensus diagnosis after each follow-up session using the Diagnostic and Statistical Manual of Mental Disorders Third Edition, Revised (1987) for dementia, and the National Institute of Neurological and Communication Disorders and Stroke-Alzheimer’s Disease and Related Disorders Association criteria [33], using the longitudinal neuropsychological tests and clinical data obtained in the BLSA [34]. We highlight that the extensive longitudinal nature of cognitive assessments in the BLSA provides higher confidence that a participant is indeed normal as opposed to having undetected incipient cognitive impairment, which would compromise the interpretation of findings. More details about data sharing to researchers can be found in the BLSA study website (http://www.blsa.nih.gov/researchers).

### Imaging protocol

Details of our image acquisition parameters have been described in [32]. Briefly, the BLSA protocol included an axial T1-weighted volumetric spoiled gradient recalled (SPGR) series (axial acquisition, TR = 35 ms, TE = 5 ms, flip angle = 45°, voxel dimensions of 0.94 * 0.94 * 1.5mm slice thickness). Regional cerebral blood flow was measured using PET with a bolus injection for each scan of 75 mCi of [^15^O] water. The images were obtained on a GE 4096 scanner, during a resting state scan with eyes open [35]. After the brain radioactivity concentration reached a threshold, one 60-second image was acquired that included 15 slices of 6.5 mm in thickness. Attenuation correction was performed using a transmission scan acquired prior to the emission scans. 

### Image pre-processing

All MR images used in this study were pre-processed using a mass-preserving shape transformation method [36]. Gray matter (GM), white matter (WM), and cerebrospinal fluid (CSF) were segmented from each skull-stripped MR brain image [37]. Each segmented brain tissue image was spatially normalized to a brain atlas (template) that was aligned with the MNI coordinate space [21,38] via HAMMER registration [39]. Then, regional volumetric maps, named RAVENS maps were generated using tissue preserving image warping [36]. Individual intracranial volume (ICV) normalization was applied on RAVENS maps to adjust global differences in intracranial size. Last, normalized RAVENS maps were down-sampled and smoothed to incorporate neighborhood information using an 8mm FWHM Gaussian filter.

[^15^O] PET-CBF image pre-processing included normalization for global activity, removal of extraneous signal scatter by thresholding the image intensity at 80% of the gray matter mean, and removal of activity in the skull, nasal sinuses, and cerebellum. PET-CBF images were rigidly registered to the corresponding participants’ MR image then deformed to the same template space based on the spatial normalization transformation parameters determined from the MRI. After registration, PET-CBF images were smoothed using a 12mm Gaussian filter.

### Components of cognitive domains and neuropsychological test battery

During each neuroimaging visit, participants completed a battery of neuropsychological tests, which were used to measure various cognitive processing abilities. Here, we focus on executive and memory processes, which are often affected early in the development of cognitive impairment and Alzheimer’s disease [26,32,40]. Table 1 lists four specific components of executive and memory function evaluated in this study, which were derived by combining the associated tests to form composite scores [26]. Specifically, composite scores were computed as the mean normalized score across each of the associated tests for each component, for each individual. Higher scores indicated better performance. 

**Table 1:** Components of cognitive domains and the involved neuropsychological tests.

**Broad Domains**	**Components**	**Combined Tests**
**Memory**	Long-Term Memory	CVLT Long Free Recall
		CVLT Long Free vs List 5
		CVLT Long Free vs Short Free
	Short-Term Memory	CVLT Short Free Recall
		CVLT List A1
		CVLT Total Recall
**Executive**	Semantic Retrieval	Boston Naming Test
		Category Fluency Total No. of Words
	Manipulation	Alpha Span
		Digit Backwards

Memory was defined as the ability to store and retrieve episodic information. Under this broad domain, short-term memory refers to storage of episodic information over brief periods of time (approximately 1-10 minutes) and was measured as a composite of short-delay free recall, first recall of list A, and total recall over five learning trials in the California Verbal Learning Test (CVLT) [27]. Long-term memory is the ability to store episodic information over longer time periods (approximately 20 minutes or more) and was measured as the composite score of long-delay free recall, the difference between long-delay free recall and free recall on the fifth trial, and the difference between long-delay free recall and short-delay free recall in the CVLT [27]. Executive processing refers to a set of control-level processes that involves searching, selecting and organizing information. Under this domain, semantic retrieval refers to the ability to recall information from semantic memory based on semantic cues, which was measured by a composite of Category Fluency Tests and the Boston Naming Test [41]. Manipulation, measured by a composite of Alpha Span and Digit Span Backwards performance, is the ability to rearrange item information in mind [42]. 

There were some participants for whom either the neuroimaging data or the performance for a specific cognitive component was not available. Thus, there were a different number of samples from each cognitive domain, *N*_*c*_, but there was still substantial overlap of participants and comparable gender ratio and age range across the four sample groups used in the regression models (Table 2). 

**Table 2:** Characteristics of participants for four components of cognitive domains.

**Domain**	**N_c_**	**Male/Female**	**Mean age in yrs (SD)**	**Age range in yrs**
**LTM**	80	46/34	69.3 (7.5)	56.2 - 85.9
**STM**	85	51/34	70.0 (7.8)	57.5 - 85.9
**Semantic Retrieval**	75	46/29	70.2 (7.3)	56.0 - 85.9
**Manipulation**	80	48/32	69.7 (7.7)	56.0 - 85.9

### Multi-modality pattern regression method

The current work builds on our previous pattern regression study of structural MRIs, which is a methodology to measure the association between brain images and continuous variables (e.g., clinical or cognitive behavioral measures) across individuals [19]. Here, we briefly outline the three basic steps of the method, which were applied separately for each cognitive domain, and point the reader to more in-depth explanations available in the previous study [19]. Note that we implemented a leave-k-out cross-validation scheme that repeated steps 1 and 2 below over all permutations of the sample for each cognitive domain with 10% of full sample size (k_c_ = 0.1*N_*c*_) used to estimate modeling errors. 

For each leave-k-out case, step 1 involved feature generation and ranking to reduce high dimensional image voxel data to a set of brain regions (features), thereby reducing computational load. Specifically, we first used a watershed algorithm, along with spatial consistency constraints, to segment the brain RAVENS maps into a number of regional voxel clusters that present similar correlations with the cognitive variable being considered. The watershed segmentation algorithm is traditionally an image segmentation approach for partitioning images into different regions according to local tissue density similarity [43]. It is able to automatically determine the number of clusters from the data, resulting in a completely unsupervised approach [44]. In addition, the watershed algorithm is known for its effective handling of boundary situations of different areas, which is advantageous for generating morphologically consistent brain regions. By adapting this process to PET images as well, a whole set of features were generated for the structural and functional modalities for each cognitive domain. Next, as in our previous study [19], we determined the correlation of each feature with cognitive performance and chose the top 3*(*N*_*c*_ *- k*_*c*_) ranking features, based on their correlation power, as the set of initial brain regions that were fed into the next step. The use of top-ranked feature sets also helped the subsequent feature selection method achieve convergence with reasonable computation cost. In step 2, we used the RVR-RFE method to optimize prediction accuracy through feature selection and model training [19]. Specifically, we used a backward selection method that removed a feature each iteration if the mean squared error (MSE) of the RVR model without that feature (applied on the *k*_*c*_ left-out validating samples) was smaller than that of the full model and was smallest amongst all other possible cases of left-out features in that iteration. Backward selection proceeded until the MSE no longer reduced with further feature removal. Next, to avoid missing informative features whose rank was low, but which performed well jointly with the top ranked features for regression, a forward feature selection method was applied that added one feature back at a time using the same MSE criteria as in backward selection. Thus, RVR-RFE integrated feature selection with RVR model building by choosing a subset of top-ranked features that optimized the performance of the RVR regressors. We highlight that, although this is essentially a sequential algorithm, the use of backward and then forward selection procedure avoids suboptimal performance, local maxima issues, and also improves computational time. To interpret the resulting RVR model within the framework of the original image modalities, we used a discriminative direction method that computes the brain region contributions based on the model that moves the predicted cognitive score closer to the actual score while minimally changing the input feature vector of the model (see 45 for more details). In this way, we computed spatial difference maps indicating the degree to which each brain region contributes to the regression prediction [19]. Thus, for each leave-k-out case of the cross-validation, a regression model was built with the optimal feature set, performance prediction and the corresponding spatial difference map that were then submitted to step 3.

For step 3 of each cognitive domain, a summary map of brain region contributions to cognitive score prediction was obtained by averaging the spatial difference maps from all leave-k-out cases. To facilitate comparison of contribution maps across the optimized models using single or combined imaging modalities, we then normalized the contribution levels to a range from 0 to 1, indexing lowest to highest predictive contributions respectively. The predicted cognitive scores were also based on averaged optimal predicted scores across all leave-k-out cases for each cognitive domain. Overall, we performed three RVR computations for each of the four cognitive domains: one RVR using MRI (white and gray matter structure) alone, one using [^15^O] PET-CBF alone, and one using both MRI and PET. To evaluate model performances, we examined the MSEs and correlation coefficients indexing the strength of agreement between the actual cognitive scores and scores predicted by the models. Since MSEs and correlation coefficients are not normally distributed, differences in cognitive score predictions between models were statistically compared using non-parametric random permutation testing [46], which were also the simultaneous tests for model differences in prediction accuracy. Specifically, the permutation tests assessed the probability of obtaining the difference between predicted scores from two models (out of 10,000 iterations), given the predicted scores from the RVR-RFE procedures.

### Ethics Statement

The study was approved by the local Institutional Review Board of the University of Pennsylvania and the National Institute on Aging. Written informed consent was obtained from all participants at each visit. 

## Results

### Predictive ability of multi-modality pattern regression model

MSEs were used to describe the performance of machine learning-based regression models (Table 3 top). As expected, the prediction accuracies (indicated by lower MSE) of regressions combining structural and functional imaging modalities were all higher than either alone. Model performances were also indexed using the correlation coefficients between the observed cognitive scores and the predicted scores based on imaging data (Table 3 bottom). Combined models generally achieved higher correlation coefficients than single modality models across all four cognitive components with all correlations being significantly greater than zero except for functional imaging with manipulation). Except for manipulation, correlations between multi-modal imaging predicted scores and actual scores were all greater than 0.4, and the correlations for functional imaging always greater than for structural imaging. For manipulation, the correlation with structural MRI alone was greater than functional imaging alone, but the combination of both structural and functional imaging modalities still improved prediction (MSE of 0.79 for structural MRI to 0.78 for both imaging modalities corresponding with correlations of 0.32 to 0.35). Importantly, statistical comparisons using non-parametric random permutation testing revealed significantly greater predictive strength of combined relative to single imaging models (p(Combined > Structural): LTM = .011, STM = .024, Semantic Retrieval = 0.013; p(Combined > Functional): LTM = .035, Semantic Retrieval = .032, Manipulation = .022) with the exception of functional imaging for STM (p(Combined > Functional) = .053) and structural imaging for manipulation (p(Combined > Structural) = .064). The latter two exceptions are consistent with the smaller correlation differences in terms of effect sizes between combined and single imaging model prediction (STM: 0.38 for functional, 0.45 for combined; Manipulation: 0.32 for structural, 0.35 for combined). Overall, the results demonstrate that models that combined modalities were more accurate than models using single imaging modalities at predicting actual cognitive performance.

**Table 3:** Mean squared errors and correlation coefficients between the predicted and measured cognitive scores by using different imaging modalities for each cognitive component; age correlation with the measured cognitive score.

	**Structural imaging**	**Functional imaging**	**Combined**	**Age**
**Cognition component**	**Mean Squared Errors: Mean ± std (95% confidence interval)**			
**LTM**	1.75 ± 0.798 (1.17 - 2.31)	1.90 ± 1.514 (0.80 - 2.97)	0.89 ± 0.462 (0.55 - 1.20)	/
**STM**	1.36 ± 0.547 (0.95 - 1.81)	0.85 ± 0.239 (0.68 - 1.05)	0.76 ± 0.212 (0.60 - 0.95)	/
**Semantic Retrieval**	1.74 ± 0.666 (0.71 - 1.74)	1.24 ± 0.786 (1.11 - 2.32)	0.62 ± 0.228 (0.35 - 0.71)	/
**Manipulation**	0.79 ± 0.421 (0.49 - 1.09)	1.75 ± 0.742 (1.22 - 2.78)	0.78 ± 0.373 (0.51 - 1.04)	/
	**Correlation Coefficients (p-value)**			
**LTM**	0.26 (0.0194)	0.31 (0.0061)	0.41 (0.002)	-0.26 (0.02)
**STM**	0.29 (0.0063)	0.38 (0.0003)	0.45 (0.0000)	-0.19 (0.08)
**Semantic Retrieval**	0.29 (0.0107)	0.32 (0.0045)	0.46 (0.0000)	-0.32 (0.048)
**Manipulation**	0.32 (0.048)	0.13 (0.2520)	0.35 (0.0016)	-0.26 (0.018)

**Figure 1:**
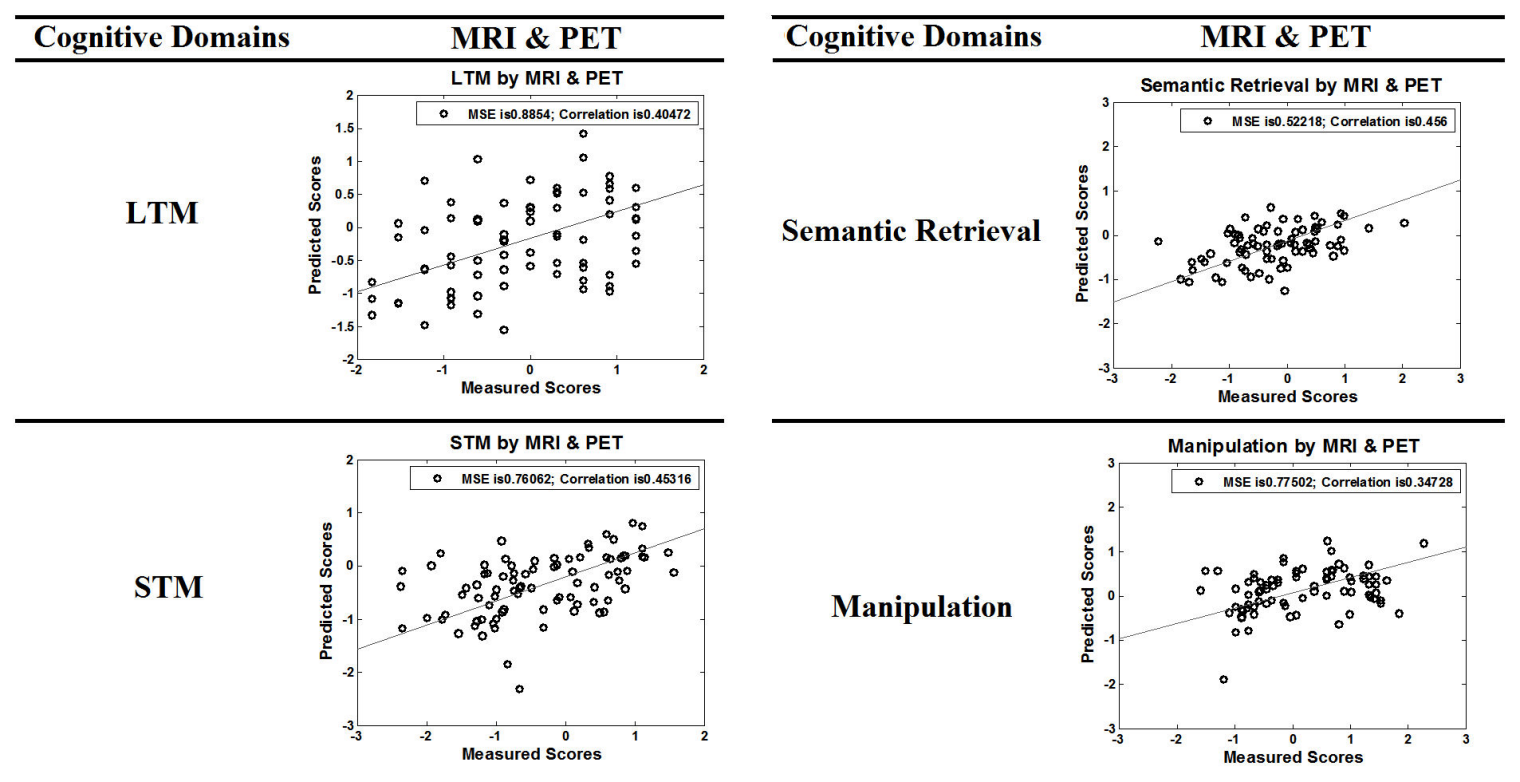
Regression results for LTM, STM, semantic retrieval and manipulation performance. The vertical axis indicates the predicted cognitive scores based on MRI and PET data, and the horizontal axis refers to the measured cognitive scores.

For visualization, Figure 1 showed scatterplots of measured and predicted cognitive scores based on the combination of MRI & PET modalities for each cognitive component. Overall, these findings indicate that our high-dimensional pattern regression method can reliably predict performance in a given cognitive domain based on information from specific brain regions, and also multiple imaging modalities provide more predictive information to build brain-cognition regression models compared to single modalities.

### Relationship of cognitive performance with age

We additionally evaluated whether the detected imaging biomarkers were more effective predictors of cognitive performance than another important factor related to cognitive decline, age. For comparison, Table 3 also lists the correlations between age and the measured scores for each cognitive domain in the last column. The machine learning-based pattern regression method showed higher correlation strengths than age in each cognitive component, supporting the notion that the combination of brain structure and function is more informative than age in accounting for cognitive performance. 

### Cognition-relevant imaging biomarkers

**Figure 2:**
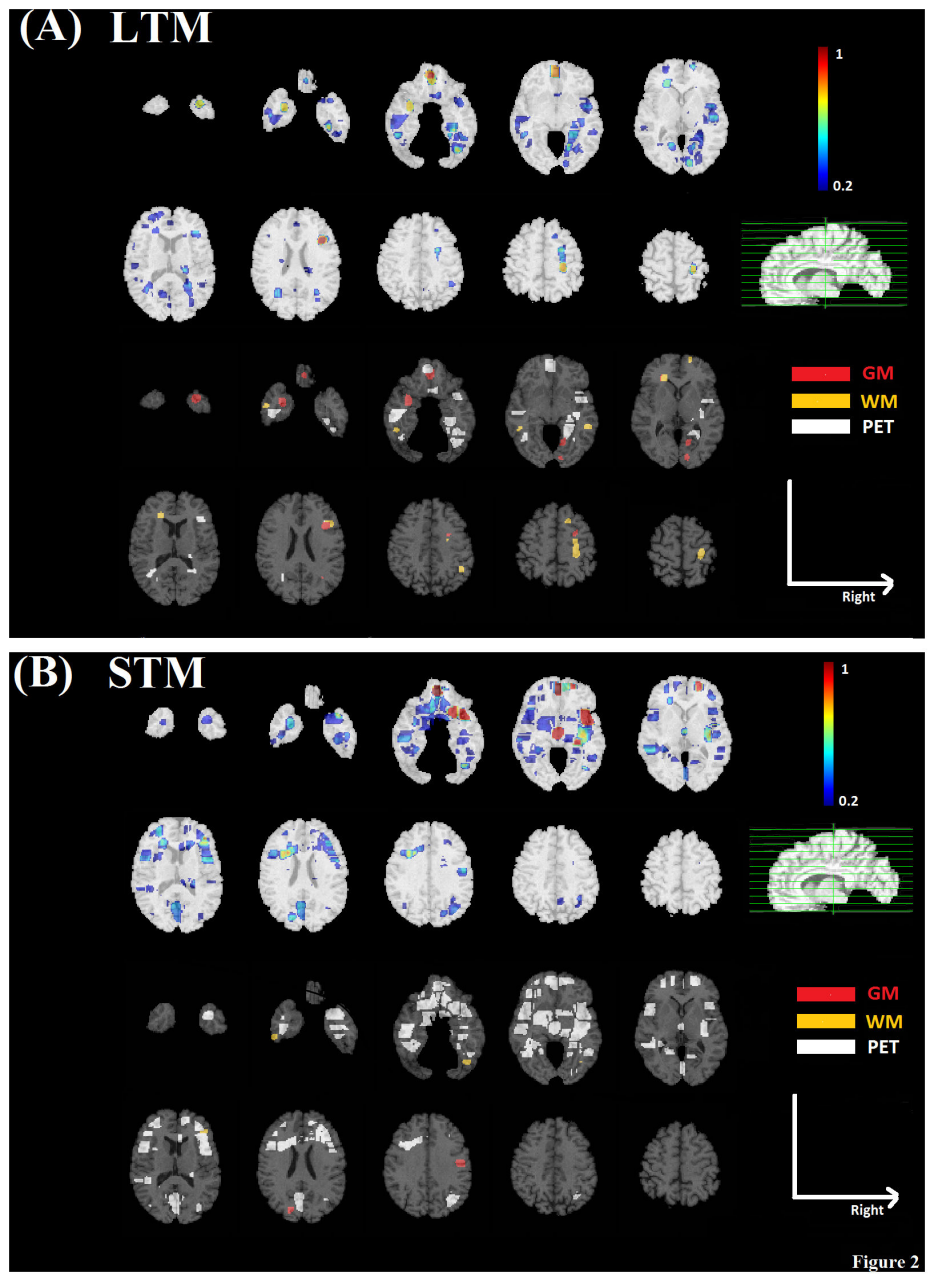
Identified brain regions related to memory functions overlaid on the template image. Contribution maps are displayed in radiological convention. A) LTM; B) STM.

**Figure 3:**
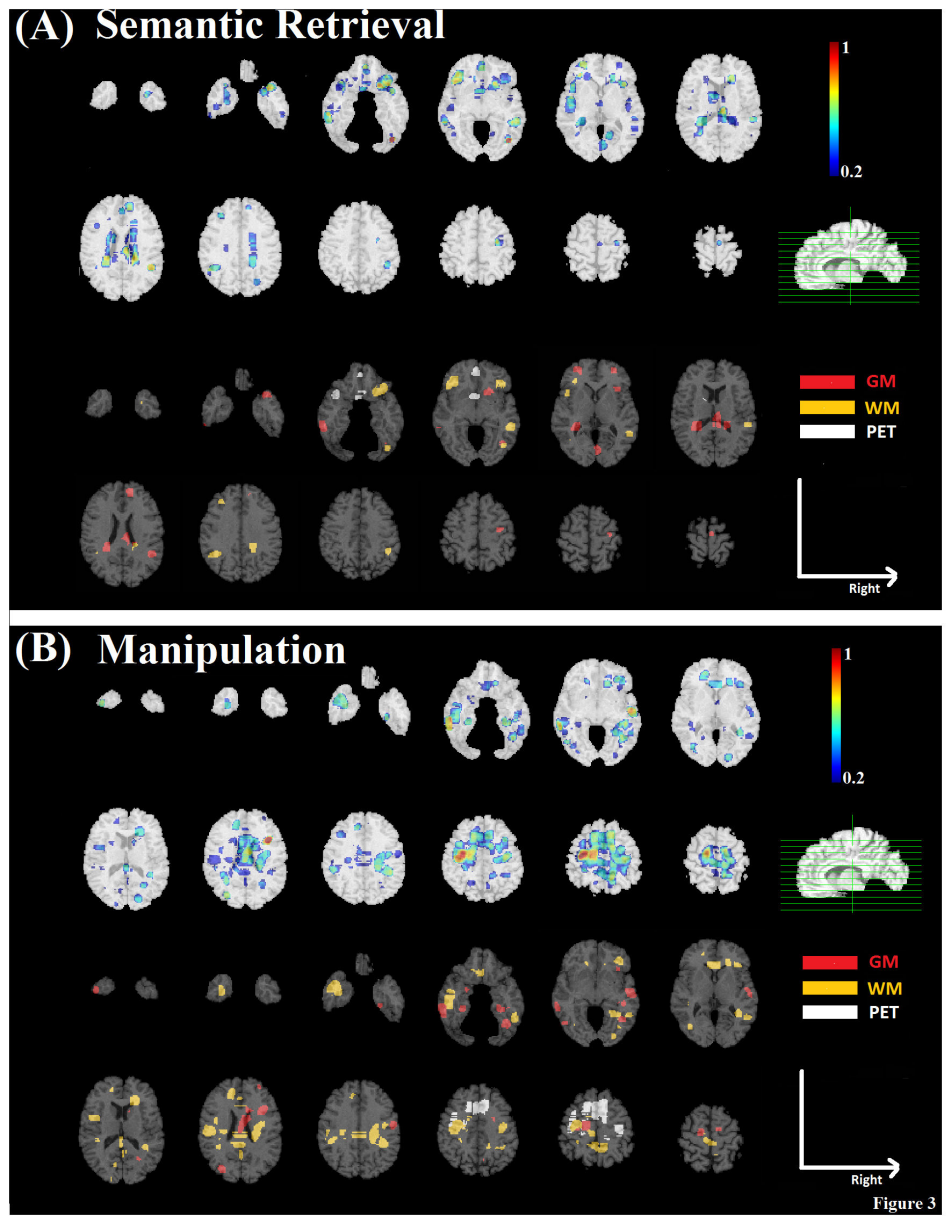
Identified brain regions related to executive processes overlaid on the template image. Contribution maps are displayed in radiological convention. A) Semantic Retrieval; B) Manipulation.

The top panels of Figures 2 and 3 show spatial difference maps indicating the combined contribution levels of brain regions identified by pattern regression analysis for each component of memory and executive functions, respectively. We highlight that maps were normalized to unit interval, with increasing degrees of contribution coded as cooler to warmer colors correspondingly. A cutoff value of 0.2 was used for the overlay maps for better visualization of the brain region contributions, however, brain regions with contribution values less than 0.2 were also used in regression modeling. In addition, the bottom panels of Figures 2 and 3 consist of categorical color maps indicating the imaging modality with the maximum contribution during pattern regression analysis for each brain region (GM: red; WM: orange; PET: white). 

As shown in Figure 2A brain regions that contributed to LTM performance prediction included the left medial orbitofrontal, and right middle frontal areas, bilateral temporal regions with extensions to the lateral occipitotemporal area, and right hippocampus and amygdala. The maximum contributions across these regions were predominantly from PET data, particularly in the medial orbitofrontal, temporal, and medial temporal areas.

Several brain regions predicting LTM were also involved in STM (Figure 2B). Specifically, STM performance was also associated with predictive contributions from medial orbitofrontal and right middle frontal regions, as well as bilateral temporal areas. Additional predictive contributions were observed in the left middle frontal and lateral orbitofrontal areas, bilateral insula, posterior cingulate, and right inferior parietal regions. Interestingly, as with LTM, maximum contributions to STM were again predominantly from PET data.

For semantic retrieval, predictive brain regions included left occipital-temporal, bilateral inferior frontal, insula, medial and lateral orbitofrontal areas, as well as right superior longitudinal fasciculus and periventricular areas (Figure 3A). Maximum contributions in the superior longitudinal fasciculus and inferior frontal regions were based on WM, but based on PET in the medial orbitofrontal area. GM was the maximum contributor in the periventricular and lateral orbitofrontal areas. 

For manipulation, predictive contributions were observed in the right inferior frontal, and insula regions, left precentral and middle frontal regions, bilateral anterior cingulate and temporal areas, as well as the left uncinate fasciculus and genu of the corpus callosum (Figure 3B). Maximum contributions in the corpus callosum, right inferior frontal region/insula were based on WM. Both WM and GM were the maximum contributors across various temporal regions. PET was the maximum contributor in the anterior cingulate region. 

### Comparison to GLM analysis

**Figure 4:**
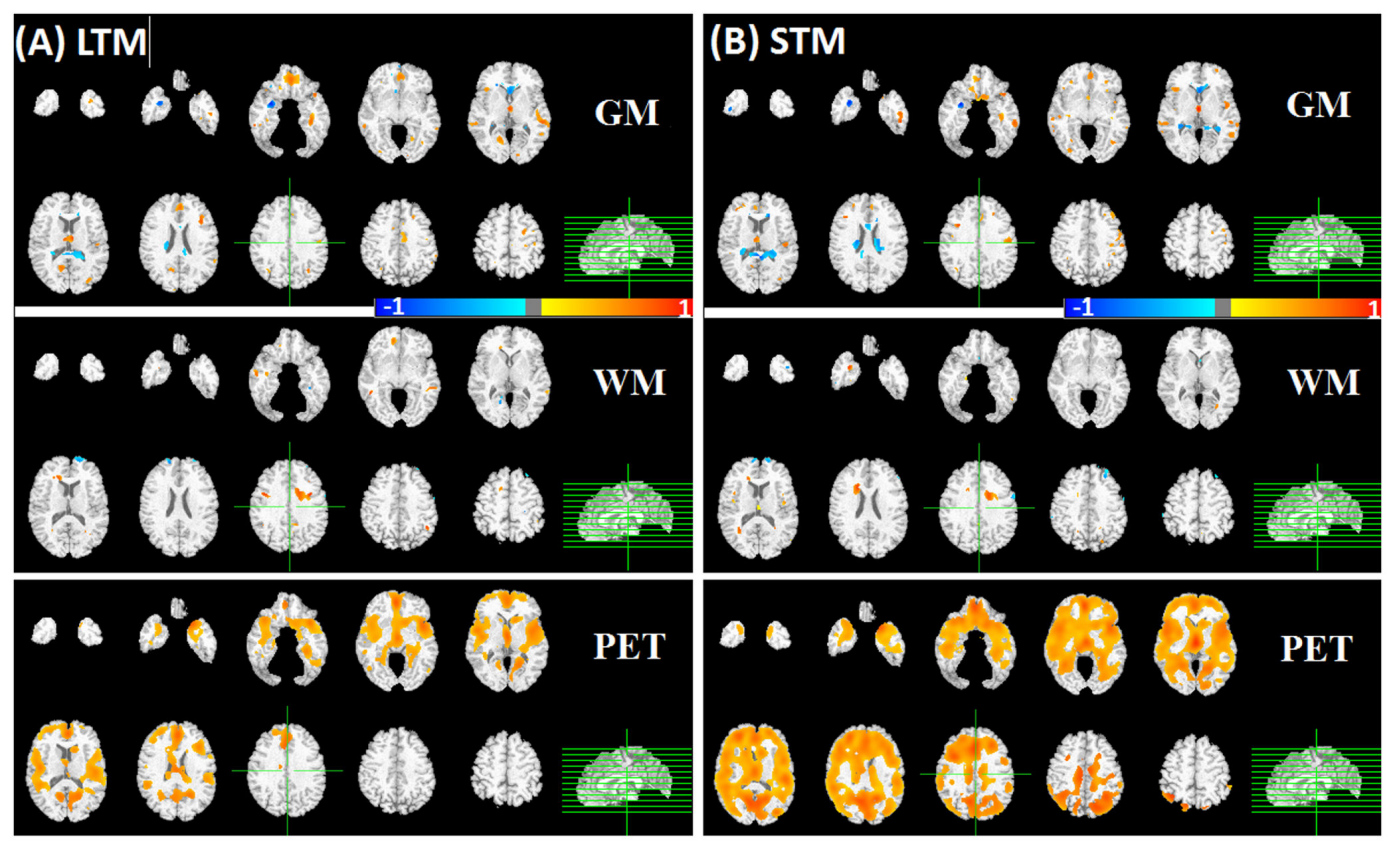
Examples of brain regions correlating with performance in the memory measures by GLM. Contribution maps are displayed in radiological convention. A) LTM; B) STM.

**Figure 5:**
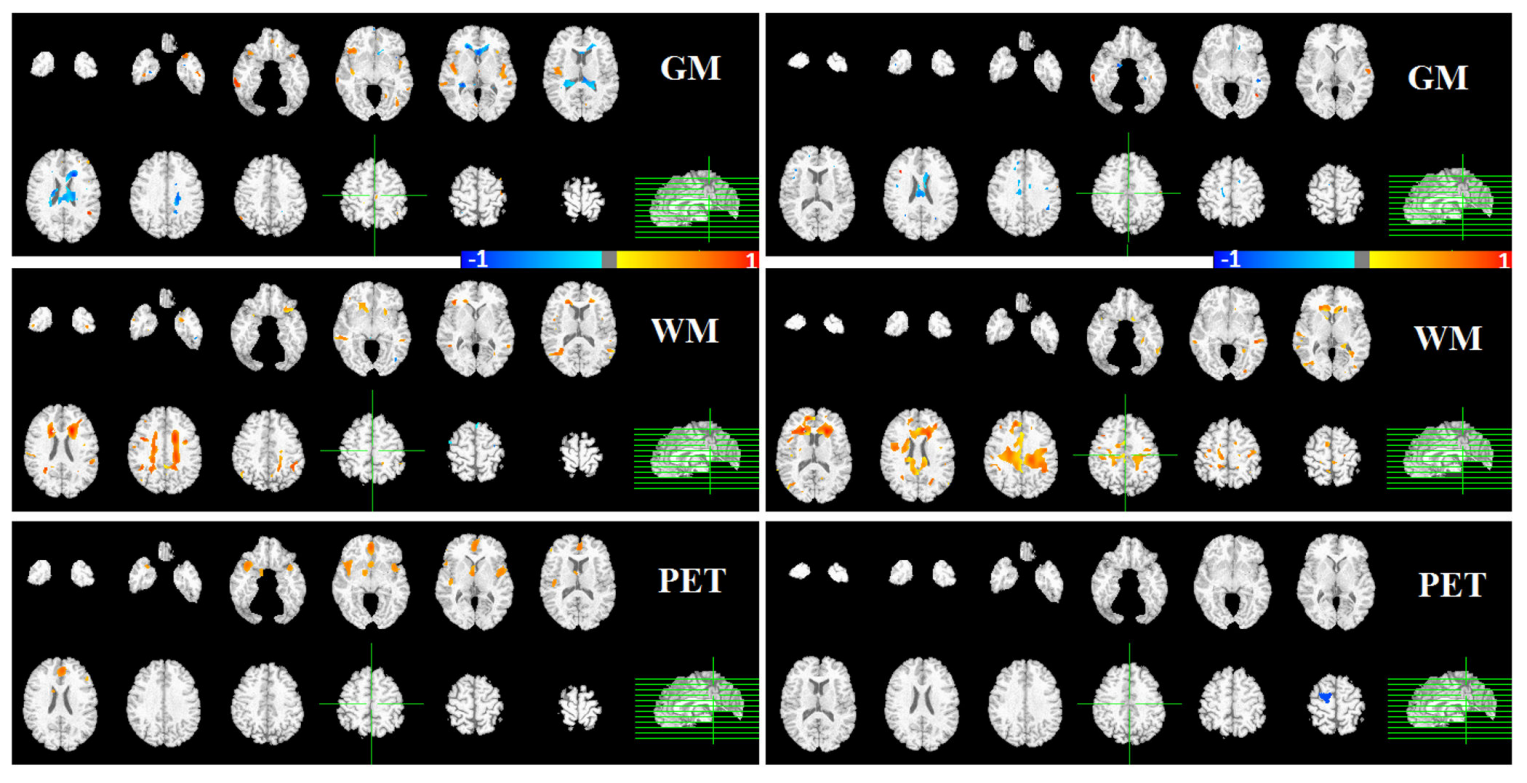
Examples of brain regions correlating with performance in the executive measures by GLM. Semantic retrieval and manipulation (left/right).

To validate the performance of our multi-modality pattern regression method, we also applied conventional GLM analysis with the same cognitive components. The GLM analyses revealed positive correlations between GM/WM/PET and cognitive performance, as shown in Figures 4 and 5. Note that unlike the contribution maps above, the color bar for GLM maps indicates both the strength and directions of the correlation with blue for high negative correlations, red for high positive correlations, and grey for zero correlation. Memory performances were strongly positively associated with the PET modality, especially for STM, which had higher and more extensive correlations than LTM (see bottom of Figure 4). These results are consistent with the multi-modality pattern regression findings that detected the greater contribution of the PET modality in comparison to WM and GM for the prediction of memory performance (Figure 2).

The GLM analysis also showed that manipulation was significantly associated with WM volumes across several regions. Similarly, the majority of brain regions detected by multi-modality pattern regression were from structural MRI, especially WM, which had the highest contribution to manipulation performance (Figure 3). However, neither GLM nor pattern regression method detected an appreciable relationship between PET and manipulation (bottom right panel in Figure 5). 

We highlight that GLM maps indicate the directions of associations separately between each imaging modality and each cognitive domain. By contrast, the contribution maps obtained from pattern regression shows the combined power of multi-modality imaging with no sign of association. Thus, the combined associations are less restricted and show simultaneous contributions across imaging modalities that could be due to a positive brain-behavior association in one modality but a negative association in another. In addition, direct comparison of the contributions across modalities is not meaningful with the GLM maps but relatively straightforward with the contribution indices used in our pattern regression method.

## Discussion

This study applied a multi-modality pattern regression method to investigate the brain imaging biomarkers related to memory and executive functions. Combined regression models for LTM, STM and semantic retrieval had similar prediction power, with regression rates of 0.41, 0.45 and 0.46 respectively (Table 3). Although these rates of around 0.45 are modest, they are in fact quite remarkable given the relatively small sample size and the large proportion of variance unaccounted for in single score measures of complex cognitive processes, which adds to prediction difficulty.

Our finding that LTM and STM involved middle and medial frontal regions, along with temporal regions and the hippocampus and amygdala (for LTM) is consistent with the existing literature on brain regions involved in episodic memory. Episodic memory tasks generally involve accessing the representations of the test items including contextual information about when and where the items were encountered. Many studies have shown that the hippocampus is an important region that forms and stores such item-context associations (e.g. [47,48]; see reviews in [49-52]). Moreover, episodic memories that are formed with hippocampal associative processes tend to be more robust over time than information encoded with rote memorization without associative tags [53]. This may account for why greater hippocampal/amygdala functional activity and structural integrity were associated with better episodic memory representations, in particular when accessing memories that may have deteriorated over time in the LTM test. For STM, it is plausible that at shorter time lags, memory traces had not deteriorated and other retrieval processes in other regions play a more important role in accounting for performance. Specifically, studies have also shown that episodic memory retrieval also involves frontal regions such as the middle and medial frontal areas observed in our study [54]. These frontal regions are involved in selecting and monitoring the contents of active memory or stored memory content in the medial temporal regions. Thus, greater activity or structural integrity in frontal regions may have accounted for better STM performance. We note that our STM and LTM measures may reflect largely overlapping processes involved in episodic memory, so that these two domains are not mutually exclusive. Nevertheless, these two measures may still also be sensitive to slightly different aspects of episodic memory. This has previously been evaluated in [26], which showed that STM and LTM performance is correlated across individuals, but still show slightly distinct relationships with other cognitive abilities, reflecting their differential sensitivity to episodic retrieval at different time scales. This notion is consistent with our finding of several overlapping regions involved in these domains along with some uniquely contributing regions.

For executive processes, we found that the inferior frontal/insula regions (around the uncinate fasciculus), superior longitudinal fasciculus and genu were associated with semantic retrieval and manipulation performance, with the maximum contribution from white-matter images. Other studies have similarly found that these white-matter regions show structural declines with age that are associated with poorer performance in tests of executive function and working memory [55-57]. These findings suggest that for the executive processes evaluated here, white-matter connectivity was of particular importance over and above gray matter volume and functional activity. Again, while there is substantial overlap, semantic retrieval may involve more rapid communication between fronto-parietal attention and control processes and other storage and representational systems in the temporal lobe. Specifically, semantic retrieval requires participants to search, select, and match as many words as possible based on semantic cues. Thus, neural connections between the frontal and temporal cortex (uncinate fasciculus), and between frontal and parietal cortex (superior longitudinal Fasciculus) are important for semantic retrieval. With manipulation, participants have to hold stimulus information in mind and, critically, apply some re-organization to the information. Thus, communication between different frontal regions might be more critical rather than searching or selecting from storage, and the white-matter connection across contralateral frontal regions (genu) might be more important.

This present study focused on modeling the continuous cross-sectional distribution of cognitive performance across individuals instead of individual longitudinal performance changes over time. It is plausible that longitudinal data may capture distinct important regional contributions toward cognitive performance [30,58]. We note as well that the use of a single 60 s PET scan may be a less than optimal measurement of regional cerebral blood flow in the present cross-sectional analysis. Thus, future work is necessary to validate our methodology for predicting individual changes based on longitudinal brain and behavioral trajectories. In addition, we note that the contribution of each neuroimaging modality to cognitive performance appears small relative to observations based on clinical samples in other studies. This is not surprising as the goal here is to derive useful information based on pre-clinical samples, rather than to replicate the more obvious differences typically observed with clinical-based samples. Nevertheless, recent directions, such as the impetus to identify early biomarkers of AD, point to the importance of combining multiple sources of diagnostic information to improve classification accuracy. Our findings thus provide further comprehensive validation of the present machine-learning methodology as a platform for developing more enhanced ways to obtain multi-modal imaging biomarkers of pre-clinical human behavioral performance.

## Conclusion and Future Work

We investigated the utility of multi-modality high-dimensional pattern regression for detecting imaging-based biomarkers of cognitive ability in a cohort of cognitively normal older adults. Our findings show that this machine learning-based pattern regression method is able to detect specific brain regions associated with different cognitive processes, while simultaneously assessing the contribution of different imaging modalities. Moreover, the combination of PET and MRI was better than each modality alone. Critically, our results suggest that functional imaging provided slightly better predictive ability than structural MRIs for memory functions, but WM modality outperformed PET when predicting executive functions. The predicted cognitive scores using our methodology could be used to identify individuals with risk of future cognitive decline from baseline images, such as in the case of when the predicted score deviate from the normal range. Future studies will evaluate imaging biomarkers of additional aspects of memory and executive function as well as the predictive ability with respect to longitudinal performance.
